# Recent Researches on the Action of Cholagogues

**Published:** 1881-05

**Authors:** H. S. Kilbourne

**Affiliations:** Ass’t Surgeon U. S. Army


BUFFALO

MEDICAL AND SURGICAL JOURNAL.

VOL. XX.—MAY, 1881.—NO. 10.

### Original Communications.

### RECENT RESEARCHES ON THE ACTION OF CHOLAGOGUES.*

BY H. S. KILBOURNE, M. D., ASS'T SURGEON U. S. ARMY.

SOME years since the medical world was stirred by the report of the Edinburgh Committee, of which Dr. Bennett was chairman. This committee reported, among other matters, that they had administered calomel to dogs with biliary fistulæ, after the physiological method, with the effect not only of not increasing the secretion of bile from the liver, but of actually diminishing it. From the results of these experiments, it was concluded that calomel had no probable effect, as a cholagogue on man. The high standing of the committee gave a force to its *dictum*, which was almost ludicrous in its effect on the minds of many of the profession. Those who had for years relied on the traditions of the fathers and the old formula of "ten and ten," for a "warm purge" as an essential preliminary in the treatment of most diseases, found themselves at sea, with the sheet anchor overboard and gone.

* Read before the Buffalo Medical Club, March 16, 1881.

Alleged abuses in the use of calomel in the army, during the civil war, induced the then Surgeon General to publish an order striking it from the supply table, on the ground that it had lost its place in rational therapeutics. In effect, this physic was thrown to the dogs. This, however, was prior to the appearance of the report of Dr. Bennett's committee, but subsequent to the publication of his treatise on the practice of medicine, which gave such an impetus to the movement against the old antiphlogistic system of medication, including blood-letting, the use of calomel, tartar emetic, etc.

After a time clinical evidence was produced to prove that the drug, when administered to healthy men, largely increased the amount of bile in the dejections, and by this the current of opinion was partly arrested and turned back. At present there is a division of opinion on the subject, and authorities are conflicting. Stillé and Maisch say: "On the whole the most probable conclusion upon this subject is expressed in these words; calomel is now a cholagogue, but diminishes the secretion of bile."

Wood says, in concluding a review of the evidence, "The conclusion seems inevitable that mercurial purgatives given to healthy persons cause the escape of large quantities of bile from the alimentary canal."

Excepting the experiments of Röhrig on curarized dogs in 1873, quoted by H. C. Wood (Therapeutics 1874, page 374), the most systematic, complete and satisfactory work in this department of therapeutics on record, is that which has recently been completed by the report of the Scientific Grants Committee of the British Medical Association, of which Dr. Rutherford is chairman.

The method of experiment is recapitulated as follows: All the experiments were performed on dogs, because of the suitable size of that animal, and for the reason that they have shown that the liver of the dog is influenced in the same way, although it may not be in like degree, by the agents which excite the biliary secretion in man.

Each animal had a full meal of flesh in the afternoon, and at nine o'clock on the following morning the experiment was begun,when, of course, the absorption of food had ceased, a condition essential for a constant secretion of bile. In every case irregular muscular movements were prohibited by repeated small doses of curare, it having been ascertained that these have no influence on the biliary secretion, nor do they prevent the manifestation of the effects of hepatic stimulants. Through an opening in the linea alba, a glass canula was inserted into the common bile duct, the crystic duct was clamped and the bile compelled to flow into the canula as fast as it was secreted.

The bile dropped into a graduated cubic centimetre measure, and a record was made of the amount secreted every fifteen minutes. Each experiment lasted the greater part of a day, at the close of which the animal was killed, and its alimentary canal examined. The various drugs were not injected into the stomach, because of its containing a very considerable quantity of viscid saliva in the fasting dog, the mucin of which is apt to envelop various medicinal agents, and thus retard their absorption. The substances were injected into the duodenum for the above reason, and also for the purpose of allowing them a fair opportunity of exciting its mucous membrane, and thus stimulating the liver, did they possess any power of so doing.

The action of mercurials on the liver was re-examined by the committee, and they report as the result, that calomel stimulates the intestinal glands, but not the liver (of the dog), and they find that mercuric chloride is a powerful hepatic stimulant, while it is a feeble intestinal stimulant.

Although calomel is an intestinal, but not a hepatic stimulant, excitement of the liver, as well as of the intestinal glands, results when mercuric chloride and calomel are administered together.

The conflicting character of the evidence on this subject is perhaps more apparent than real, owing to a confusion of terms. The classification of cholagogues has disappeared from many systematic treatises on therapeutics.

What is a cholagogue? The committee do not find that the term has a precise meaning, but that it is applied to any substance which increases the flow of bile, whether by augmenting the secretion, or by exciting contraction of the bile ducts and gall bladder. They have to do only with the secretion of the bile, and employ the term hepatic stimulant for those substances which increase it.

We are indebted to this English Committee for an advance in the knowledge of the action of several of our indigenous remedies included in the scope of their investigation. These, among a number of other articles of our native materia medica, have been neglected by the profession, because their properties are not fully known and because, perhaps, many are discredited by being in the hands of quacks and empirics. Of this class the one best known is podophyllin, it is in common use and its cholagogue properties are acknowledged as well as its irritant effect on stomach and bowels. The committee has shown that if the dose of this drug be too large the secretion of bile is not not increased (in the dog), a fact full of suggestiveness to the therapeutist.

Euonymin (from the euonymus atropurpureous, wahoo) was found to be a powerful hepatic stimulant and less of an intestinal irritant than podophyllin.

Sanguinarin (from sanguinaria canadensis, blood root), they find to be a powerful hepatic stimulant; it also stimulates the intestines less than podoplyllin.

Leptandrin (from leptandria virginica, Culver's root) was found to be a hepatic stimulant of moderate power, but a feeble intestinal stimulant.

Baptisin (from baptisia tinctoria, wild indigo) they find to be a hepatic stimulant, and also an intestinal stimulant of moderate power (dose for a man, from one to five grains).

Phytolaccin (from phytolacca decandria, poke root) they find to be a hepatic stimulant of considerable power. It also slightly stimulates the intestinal glands (dose for a man, from one to three grains).

Hydrastin (from hydrastis canadensis, golden seal) is reported to be a hepatic stimulant of moderate power, and a feeble intestinal stimulant (dose for a man, one to two grains).

Juglandin (from juglans cineria, butternut) was found to be a moderately powerful hepatic, and a mild intestinal stimulant. This drug is regarded as especially worthy of the attention of physicians.

Iridin* (from iris versicolor, blue flag) they find to be a powerful hepatic stimulant. It stimulates the intestine, but less than podophyllin (dose for a man from two to five grains).

Taraxacum was found to be a very feeble hepatic stimulant. This completes the last of native drugs selected from the fifty or more different articles experimented on by this notable committee.

As a sequel to the ascertained effects of these substances in the hepatic secretion of the dog, the committee say: "Although we must leave to our medical brethren the task of testing on the human subject the effects of baptism, sanguinarin, phytollaccin, etc., and will doubtless be of service if we here recount our ex-experience in the use of enonymin and iridin. As yet we have found four grains of iridin, made into a pill with confection of roses, and taken at bedtime, a certain remedy for biliousness. It produces no disagreeable sensations, and on awakening in the morning, the yellow tongue is found to be clean, and the headache and malaise gone.

As iridin, though a powerful hepatic, is not a powerful intestinal stimulant (cathartic), it is well to give in the morning an ordinary saline aperient, such as Püllna water, or some other. But iridin, though an agreeable remedy at the time, leaves a somewhat depressed effect, and it probably should not be taken oftener than once a week or so.

Enonymin is a hepatic stimulant in man, as in the dog. Two grains of it in pill with conf. rosæ, and taken at night, seem to

*These substances are not alkaloids, but impure preparations made by precipitating a tincture in water and collecting the precipitate, which probably contains the active principle of the drugs associated with various impurities. The articles used by the committee in these experiments were obtained from Keith & Co. of New York.

be as efficient a remedy for biliousness as iridin; if the dose be not too great, it leaves no depression. As it is a feeble intestinal stimulant, it is well to follow it in the morning with a saline aperient.

I have been much struck with the success of enonymin in functional hepatic derangements in several persons who have taken nearly all the commonly used cholagogues with varying and often very limited success.

I have no doubt that in consequence of our experiments, enonymin will be a universally employed hepatic stimulant."

Among the other articles subjected to experiments by the committee were the saline purgatives, with the following results:

Magnesium sulphate is an intestinal, but not a hepatic stimulant.

Rochelle salt is a feeble hepatic, but a powerful intestinal stimulant.

Potassium sulphate is a hepatic and intestinal stimulant of considerable power, its action on the liver is uncertain, probably owing to its insolubility.

Sodium sulphate is a hepatic stimulant of considerable power; it also stimulates the intestinal glands.

Sodium phosphate is a powerful hepatic and a moderately powerful intestinal stimulant.

Should these effects be obtained in the human subject, it seems to the writer that the latter salt will to a great extent supplant the use of magnesium sulphate; the dose is about the same, the sodium salt is less soluble than the other, requiring at 68° Fahr. seven times its weight of water, but at 86° it dissolves in 2½ time its own weight. It has a cooling saline, but not disagreeable taste instead of the nauseous bitter of the other salt. But its chief claim to favor would be that it added cholagogue to its cathartic powers, which are lacked by the other.

The following named cathartics were examined and found to be hepatic stimulants of varying powers in the order named: Aloes, jalap, colocynth, rhubarb, senna, croton oil, and scammony—the first named possessing considerable and the last, slight powers.

The following named remedies were examined and found to have no effect on the liver of the dog: Castor oil, gamboge, sulphate of manganese.

The committee report the following named substances experimented on to be powerful hepatic stimulants but without cathartic effects: Sodium salycilate, sodium benzoate, and ammonium benzoate.

This is important as showing that the action of drugs on the liver may be entirely independent of their effects on the bowels.

Further than this it is shown by these experiments that, while slight purgation by a purely intestinal irritant, scarcely at all affected the secretion of bile, powerful purgation produced a marked depression of the secretion.

Ipecacuanha is a powerful hepatic stimulant. It increases slightly the secretion of intestinal mucus; but has no other apparent stimulant effect on the intestine. The bile, secreted under the influence of ipecac, has the normal composition.

Dilute nitro-hydro-chloric acid is a hepatic stimulant of considerable power.

Ammonii chloride stimulates the intestinal glands, but not the liver.

Morphia has no appreciable effect on the secretion of bile.

Pure dilute alcohol does not affect the biliary secretion.

Japorandi is a feeble hepatic stimulant.

Calabar bean stimulates the liver, but not powerfully, unless it be given in very large doses.

Of all the drugs examined, but one, acetate of lead, appeared to have a directly depressing action on the secretion of the liver.

While the results of these experiments can not be accepted as conclusive as regards the action of these remedies on many, it appears reasonably certain that many of them affect the higher animals in the same way as man, and in no other way. The experiments furnish a fine example of the physiological method or research in the department of therapeutics, as contrasted with the clinical.

The first method has its limitations, but within these it has more precision, and is more in accord with the methods of science than the other. The amount of clinical evidence as to the action of the mercurials, for example, is so enormous that, as Wood remarks, it seems scarcely possible that it is true. But if we accept the evidence of these researches, it must be admitted as probable that the mercurial in more common use has not the peculiar power over the function of the liver, which has been claimed for it heretofore.

Here we take leave of this interesting subject, and refer the reader, who may desire further acquaintance with it, to the report of the committee, which may be found *in extenso* in the files of the British Medical Journal for the years 1875, '78, '79.

The work done by Dr. Rutherford and his colleagues, it seems to us is worthy of all praise. The amount of time, skill, patience, and accurate thought expended on this series of experiments can only be fully appreciated by those who know what is required for a complete equipment for such enterprises.

